# Effects of Perioperative Probiotics and Synbiotics on Pancreaticoduodenectomy Patients: A Meta-Analysis of Randomized Controlled Trials

**DOI:** 10.3389/fnut.2021.715788

**Published:** 2021-08-13

**Authors:** Gang Tang, Linyu Zhang, Jie Tao, Zhengqiang Wei

**Affiliations:** ^1^Department of Gastrointestinal Surgery, The First Affiliated Hospital of Chongqing Medical University, Chongqing, China; ^2^Department of Clinical Medicine, Chongqing Medical University, Chongqing, China

**Keywords:** alternative therapy, microorganisms, postoperative infection, pancreaticoduodenectomy, probiotics

## Abstract

Post-pancreaticoduodenectomy infections cause mortality, morbidity, and prolonged antibiotic use. Probiotics or synbiotics may be advantageous for preventing postoperative infections, but their benefits on pancreaticoduodenectomy outcomes are controversial. This study evaluated the efficacy of probiotics and synbiotics in pancreaticoduodenectomy. The Embase, Web of Science, PubMed, and Cochrane Library databases were comprehensively searched for randomized controlled trials (RCTs) that evaluated the effects of probiotics or synbiotics on pancreaticoduodenectomy as of April 16, 2021. Outcomes included perioperative mortality, postoperative infectious complications, delayed gastric emptying, hospital stay length, and antibiotic-use duration. The results were reported as mean differences (MDs) and relative risks (RRs) with 95% confidence intervals (CI). Six RCTs involving 294 subjects were included. Probiotic or synbiotic supplementation did not reduce the perioperative mortality (RR, 0.34; 95% CI, 0.11, 1.03), but reduced the incidences of postoperative infection (RR, 0.49; 95% CI, 0.34, 0.70) and delayed gastric emptying (RR, 0.27; 95% CI, 0.09, 0.76) and also reduced the hospital stay length (MD, −7.87; 95% CI, −13.74, −1.99) and antibiotic-use duration (MD, −6.75; 95% CI, −9.58, −3.92) as compared to the controls. Probiotics or synbiotics can prevent infections, reduce delayed gastric emptying, and shorten the hospital stay and antibiotic-use durations in patients undergoing pancreaticoduodenectomy. These findings are clinically important for promoting recovery from pancreaticoduodenectomy, reducing the incidences of antibiotic resistance and iatrogenic infections, and reducing the medical burden.

## Introduction

Pancreatoduodenectomy is the primary treatment for pancreatic and periampullary carcinomas and is a complex, high-risk procedure (1, 2). Advances in perioperative management and surgical techniques have reduced the associated mortality rate. However, the postoperative complication rate remains high; up to 60% of the patients experience complications, mainly comprising postoperative infections and delayed gastric emptying (1, 3–5). Postoperative complications lead to longer hospital stays, a higher financial burden, and an increased risk of death (1, 6, 7). Therefore, prevention is crucial for improving pancreaticoduodenectomy outcomes.

Probiotics are microorganisms that are beneficial to the human body when supplemented in appropriate amounts (8). They have anti-inflammatory, anti-tumor, and antioxidant properties, and have been used to treat antibiotic-associated diarrhea, steatohepatitis, diabetes, inflammatory bowel disease, and necrotizing enterocolitis (9–11). Prebiotics are substances (such as inulin and fructooligosaccharides) that promote the growth of beneficial gut microorganisms (12). Synbiotics are formulations that combine probiotics with prebiotics (8). The close relationship between probiotics/synbiotics and gastrointestinal microorganisms has attracted increasing attention in recent years. Probiotics can stabilize the intestinal barrier, inhibit the growth of harmful bacteria in the intestinal tract, and regulate the local and systemic immunity; these effects may help reduce the risk of intestinal bacterial translocation and infection. Prebiotics can stimulate the growth of beneficial bacteria in the gut, and play a synergistic role with probiotics (10, 13, 14). The types and dosages of probiotics or synbiotics used for the prevention of postoperative infections vary greatly, with most studies using lactic acid bacteria supplements alone or in combination with some prebiotics (10, 14). Numerous studies have reported the beneficial effects of probiotics and synbiotics on abdominal surgery outcomes (15–18). A meta-analysis showed that probiotic and synbiotic supplementation reduced the rate of infection-based complications as well as the hospital stay length following gastrointestinal surgery (19). However, the effect of probiotics or synbiotics on the outcomes of pancreaticoduodenectomy remains controversial. Rayes et al. (20) reported the first clinical study to show that synbiotics reduced the risk of complications associated with pancreaticoduodenectomy and antibiotic usage. However, Diepenhorst et al. (21) found that probiotic supplementation did not reduce the incidence of pancreaticoduodenectomy-associated complications. Since then, several studies (5, 22, 23) have investigated the effects of probiotics and synbiotics on post-pancreaticoduodenectomy infections; however, to the best of our knowledge, no systematic review or meta-analysis has summarized the current evidence.

Probiotics and synbiotics may represent potential strategies for improving the short-term clinical outcomes of pancreaticoduodenectomy. This study aimed to clarify the efficacy of probiotics and synbiotics in treating post-pancreaticoduodenectomy complications by conducting a meta-analysis on patients who underwent pancreaticoduodenectomy.

## Methods

### Search Strategy

We successfully registered this meta-analysis on PROSPERO (registration no. CRD42021249301). Electronic searches were conducted on the Embase, Web of Science, PubMed, and Cochrane Library databases with no filters to identify relevant literature published from inception to April 16, 2021. The search terms were (pancreaticoduodenectomy OR whipple OR pancreatoduodenectomy) AND (synbiotics OR synbiotic OR probiotics OR prebiotics OR prebiotic OR probiotic) (Table 1). Reference lists of related reviews were also searched.

**Table 1:** Electronic search strategy.

**Database**	**Search term (establish to April 16, 2021)**	**Number**
PubMed (All fields)	#1: synbiotics OR prebiotics OR probiotics OR synbiotic OR prebiotic OR probiotic	#1: 38291
	#2: Pancreaticoduodenectomy OR Whipple OR Pancreatoduodenectomy	#2: 18089
	#3: #1 AND #2	#3: 14
Embase (All fields)	#1: synbiotics OR prebiotics OR probiotics OR synbiotic OR prebiotic OR probiotic	#1: 55731
	#2: Pancreaticoduodenectomy OR Whipple OR Pancreatoduodenectomy	#2: 30554
	#3: #1 AND #2	#3: 26
Cochrane library trials (All fields)	#1: synbiotics OR prebiotics OR probiotics OR synbiotic OR prebiotic OR probiotic	#1: 8381
	#2: Pancreaticoduodenectomy OR Whipple OR Pancreatoduodenectomy	#2: 1226
	#3: #1 AND #2	#3: 13
Web of science (All fields)	#1: synbiotics OR prebiotics OR probiotics OR synbiotic OR prebiotic OR probiotic	#1: 54860
	#2: Pancreaticoduodenectomy OR Whipple OR Pancreatoduodenectomy	#2: 20371
	#3: #1 AND #2	#3: 20

### Study Selection

Studies that met the following criteria were included: (I) were a randomized controlled trial (RCT; any existing language), (II) included patients of any age undergoing pancreaticoduodenectomy, (III) intervention with probiotics or synbiotics (any dose, species, and strain), (IV) the control group received the standard treatment or a placebo, and (V) the outcomes included any of the following: infection, postoperative mortality, duration of antibiotic usage, and hospital stay length. Reviews, case reports, letters, abstracts, duplicate studies, and animal studies were excluded.

### Data Extraction

Data, including the first author, year, study type, sample size, age, sex, primary disease, surgery type, number of treatment days, intervention type, and control groups, were extracted from each study. If any data could not be obtained from a study, the corresponding author of that study was contacted in an attempt to do so.

### Quality Assessment

Quality assessment was based on the bias risk assessment tool provided in the Cochrane Handbook, which includes the following seven domains: (I) randomization, (II) allocation blinding, (III) participant and operator blinding, (IV) detection blinding, (V) incomplete data, (VI) selective reporting, and (VII) other biases. Literature retrieval, study selection, data extraction, and quality assessment were performed independently by two authors (Gang Tang and Linyu Zhang). If there was a disagreement between the authors, it was discussed and resolved with the third author (Jie Tao).

### Statistical Analysis

The mean differences (MD) with 95% confidence intervals (Cis) were calculated for continuous data, while the relative risks (RRs) were calculated for dichotomous variable data (24). The *I*^2^ statistic was used to assess the magnitude of heterogeneity between the studies: when *I*^2^ was >50%, the random-effects model was selected. Otherwise, the fixed-effects model was selected (25). For result robustness, the 1-study exclusion test was performed to investigate the influence of each study on the total effect size. Subgroup analysis was performed by the intervention type (probiotics or synbiotics). The Egger's test was performed using Stata 12.0 (Stata Corp., College Station, TX, USA) to assess potential publication bias. All statistical analyses were performed using Review 5.3 (The Nordic Cochrane Centre, The Cochrane Collaboration 2014; Copenhagen, Denmark). *P* < 0.05 was considered significant.

## Results

### Selected Studies

**Figure 1:**
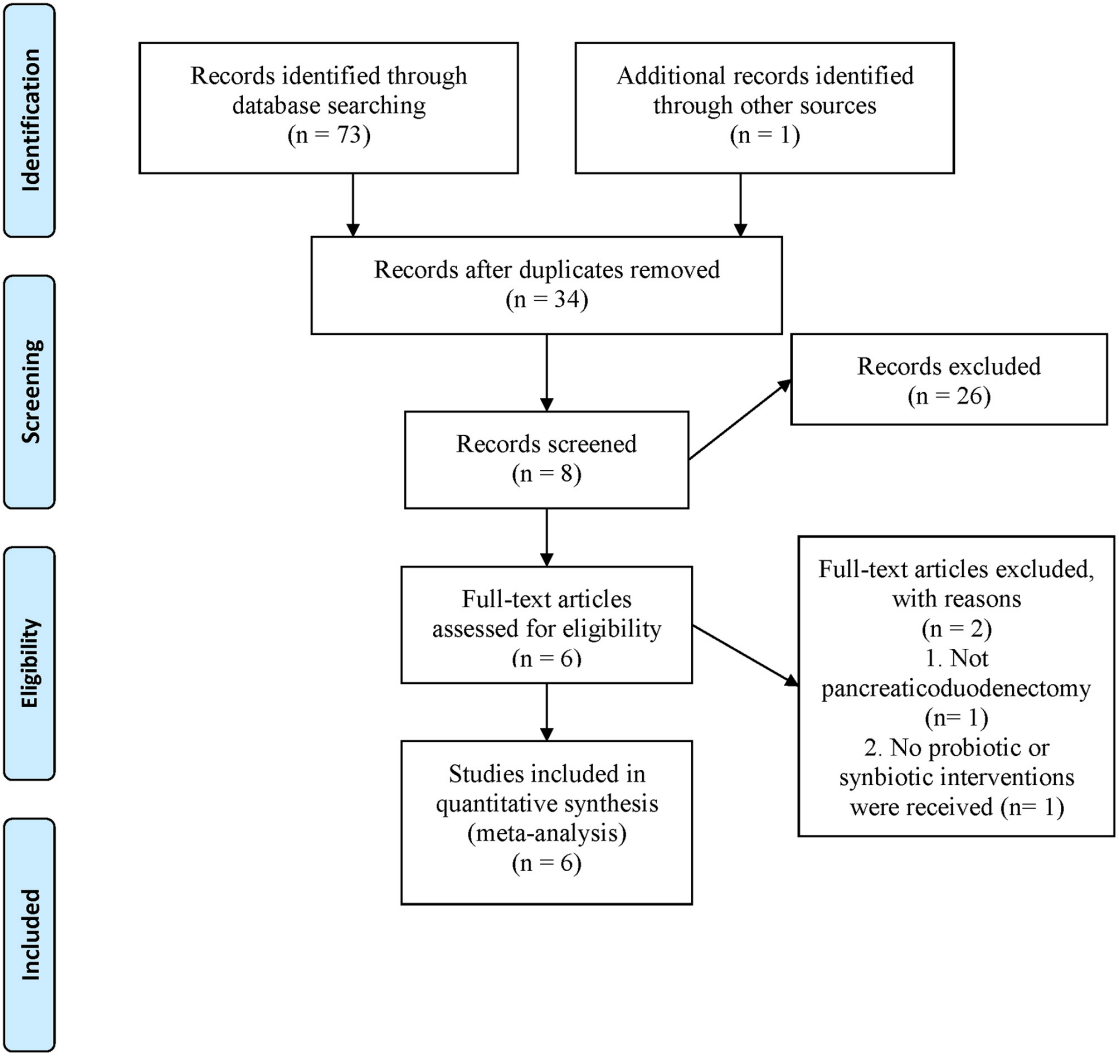
Flow chart of literature search and screening.

The search yielded 74 records; 40 duplicate studies were excluded, and the titles and abstracts of the remaining 34 articles were screened. Twenty-six reports did not meet the inclusion criteria and were excluded; the remaining eight went through a full-text evaluation. Finally, six RCTs (5, 20–23, 26) were included for analysis (Figure 1).

### Study Characteristics

Between 2007 and 2021, six studies were published with 294 total participants (147 in the intervention group and 147 in the control group). Three studies (5, 21, 26) used only probiotics, and three used synbiotics (20, 22, 23). *Pediacoccus pentosaceus, Leuconostoc mesenteroides, Lactobacillus paracasei, Lactobacillus plantarum, Enterococcus faecealis, Clostridium butyricum, Bacillus mesentericus, Lactobacillus acidophilus, Lactobacillus rhamnosus, Lactobacillus casei, Bifidobacterium bifidum, and Bifidobacterium breve strain* were used as probiotics, and inulin, pectin, fructooligosaccharides, betaglucan, resistant starch and galacto-oligosaccharide were used as prebiotics (Table 2).

**Table 2:** Characteristics of six eligible studies.

**Study**	**Type of study**	**Sample**	**Age**	**Male**	**Primary Disease**	**Type of surgery**	**Intervention group**	**Control group**	**Treated days (pre + post-surgery)**
Rayes et al. (20)	DB, RCT	I: 40 C: 40	59	45	Chronic pancreatitis, cancer	PPPD	Each dose of the combination contains: 10^10^ Pediacoccus pentosaceus 5 33:3 (dep.nr LMG P-20608), Leuconostoc mesenteroides 77:1 (dep.nr LMG P-20607), Lactobacillus paracasei subspecies paracasei F19 (dep.nr LMG P-17806), and Lactobacillus plantarum 2362 (dep.nr LMG P-20606), 2.5 g of each betaglucan, inulin, pectin, and resistant starch, totally 10 g per dose (one dose twice a day)	SC	1 day preoperative + postoperative day 1–8
Nomura et al. (26)	RCT	I: 32 C: 32	69	34	Pancreaticobilliarty disease	PD	Enterococcus faecealis T-110, Clostridium butyricum TO-A, Bacillus mesentericus TO-A, totally 6 × 10^7^ CFU (daily)	SC	3–15 days preoperative + until discharge
Diepenhorst et al. (21)	RCT	I: 10 C: 10	64	10	Periampullary or ampullary pancreatic cancer	PPPD	Ecologic 641 consisting of six probiotic strains (3 g twice a day)	SC	7 days preoperative + postoperative day 1–7
Sommacal et al. (22)	DB, RCT	I: 23 C: 23	60	N	Periampullary cancer	PD	Lactobacillus acidophilus 10, 1 × 10^9^ CFU, Lactobacillus rhamnosus HS 111, 1 × 10^9^ CFU, Lactobacillus casei 10, 1 × 10^9^ CFU, Bifidobacterium bifidum, 1 × 10^9^ CFU + fructooligosaccharides 100 mg (twice daily)	Placebo	4 days preoperative + postoperative day 1–10
Yokoyama et al. (23)	RCT	I: 22 C: 22	65	12	Chronic pancreatitis, cancer	PD	4 × 10^10^ living Lactobacillus casei strain Shirota; 1 × 10^10^ living Bifidobacterium breve strain Yakult; and 15 g of galacto-oligosaccharide (daily)	SC	7 days preoperative + postoperative day 1–14
Folwarski et al. (5)	RCT	I: 20 C: 20	62	26	Pancreatitis, cancer	PPPD	One capsule containing L. rhamnosus GG 6 million colony forming units (CFU) (every 12 hours)	SC	Postoperative day 1–30

*CFU, colony forming units; C, Control group; DB, Double blind; I, Intervention group; GOS, galacto-oligosaccharides; PD, pancreatoduodenectomy; PPPD, pylorus-preserving pancreatoduodenectomy; N; not available; RCT, randomized controlled trial; SC, standard care*.

### Quality Assessment

**Figure 2:**
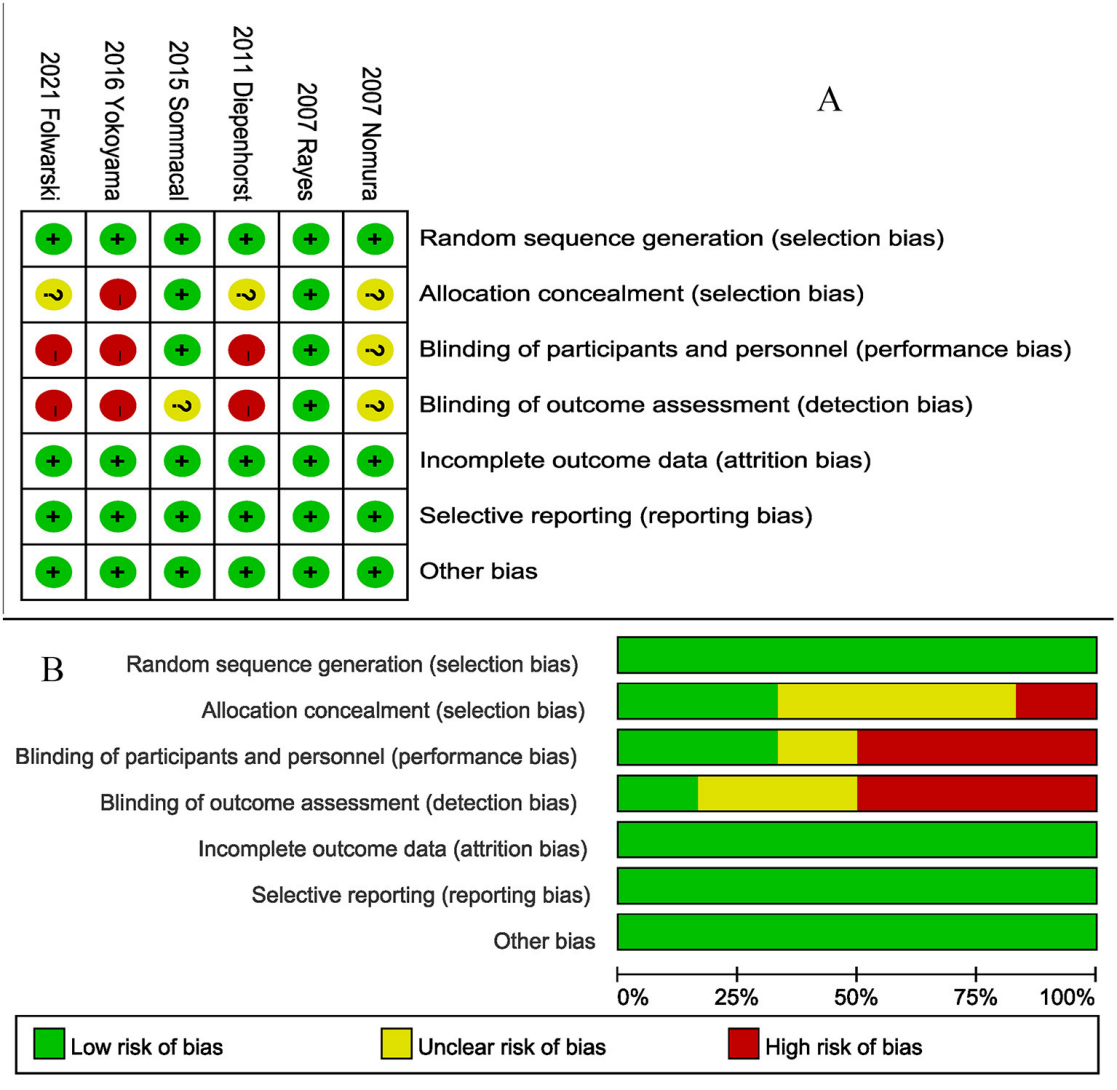
Risk of bias for each included study. **(A)** Risk of bias summary. **(B)** Risk of bias graph.

All studies (5, 20–23, 26) described their specific random assignment methods (Figure 2). Two studies used double-blind designs (20, 22), and one study (20) reported a blinded method for evaluating results. Two studies (20, 22) appropriately hid the randomization scheme. Incomplete outcome data, selective reporting, and other bias sources in all studies were assessed as a low bias risk.

### Meta-Analysis

#### Mortality

**Figure 3:**
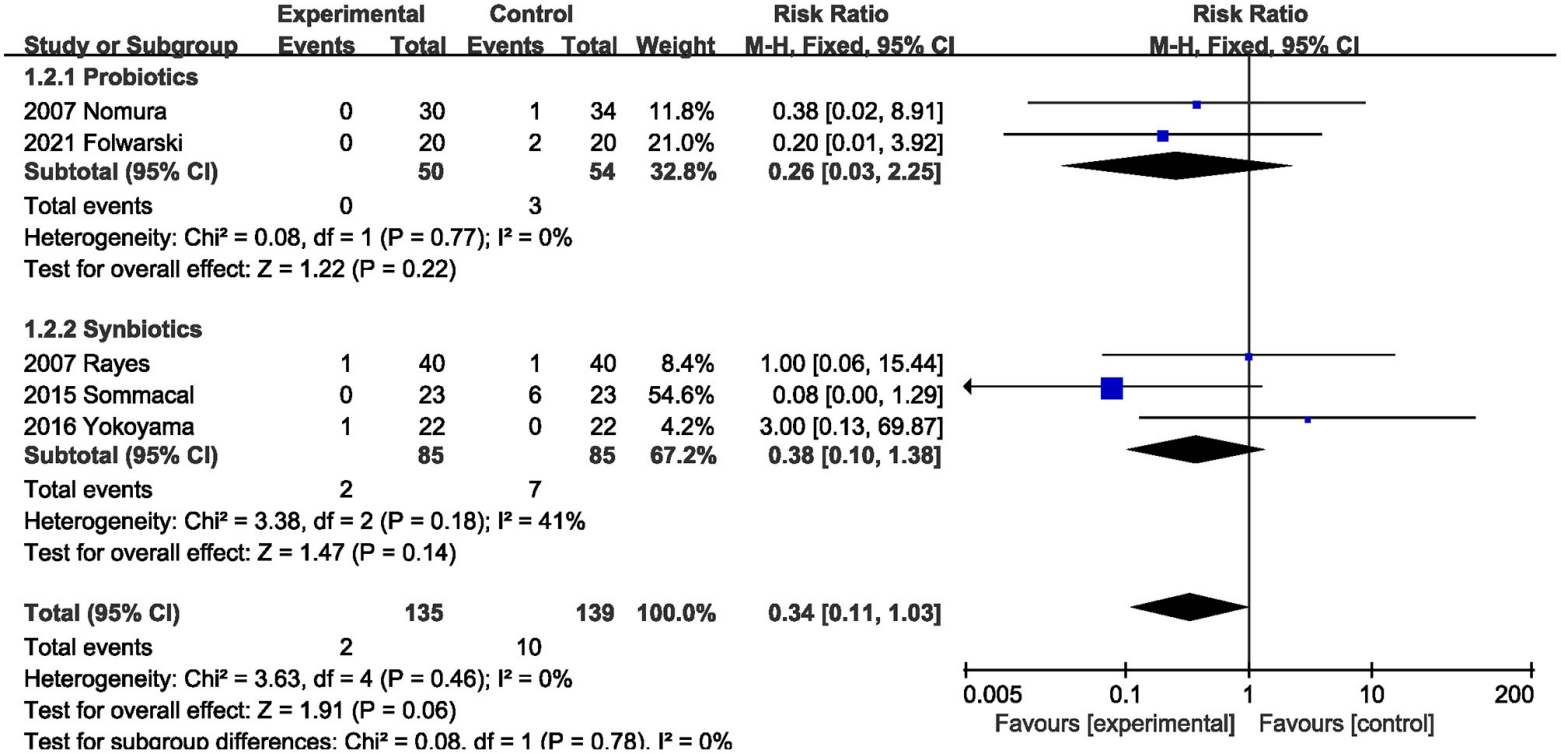
Effect of probiotics or synbiotics on perioperative mortality.

Five RCTs (5, 20, 22, 23, 26) reported on perioperative mortality. Probiotics and synbiotics did not reduce perioperative mortality compared with the control group (RR, 0.34; 95% CI, 0.11, 1.03; *P* = 0.06; *I*^2^ = 0) (Figure 3). The subgroup analysis results also showed that probiotics alone (RR, 0.26; 95% CI, 0.03, 2.25; *P* = 0.22) or synbiotics (RR, 0.38; 95% CI, 0.10, 1.38; *P* = 0.18) did not reduce perioperative mortality.

#### Postoperative Infection Complications

**Figure 4:**
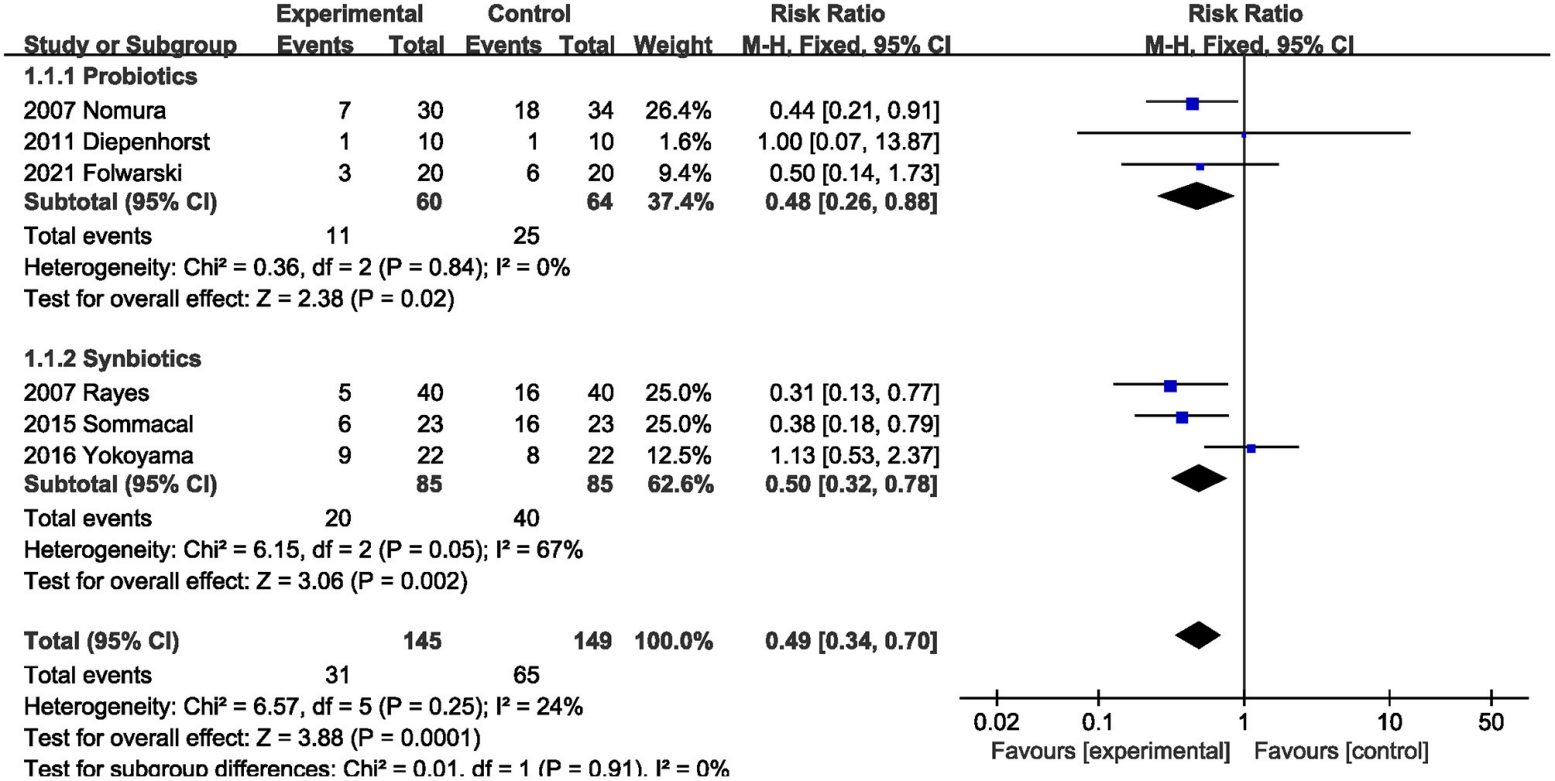
Effect of probiotics or synbiotics supplementation on the postoperative infection incidence.

All studies (5, 20–23, 26) reported postoperative infections. Pooled data showed that supplementation with probiotics or synbiotics significantly reduced the postoperative infection incidence (RR, 0.49; 95% CI, 0.34, 0.70, *P* = 0.0001), with no heterogeneity (*I*^2^ = 24%, *P* = 0.25; Figure 4) among the studies. Additionally, subgroup analysis showed that probiotics alone (RR, 0.48; 95% CI, 0.26, 0.88, *P* = 0.02) and synbiotics (RR, 0.50; 95% CI, 0.32, 0.78, *P* = 0.002) significantly reduced the postoperative infection risk.

#### Delayed Gastric Emptying

**Figure 5:**
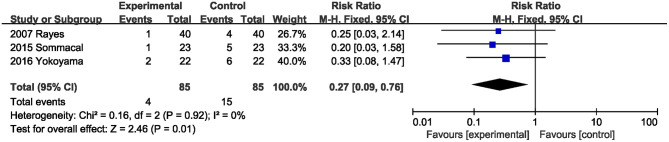
Effect of probiotics or synbiotics supplementation on the incidence of delayed gastric emptying.

Three studies (20, 22, 23) with 170 total subjects described the effects of probiotics or synbiotics on delayed gastric emptying. Compared with the control subjects, probiotics and synbiotics significantly reduced the incidence of delayed gastric emptying (RR, 0.27; 95% CI, 0.09, 0.76; *P* = 0.01; *I*^2^ = 0) (Figure 5).

#### Length of Hospital Stay

**Figure 6:**
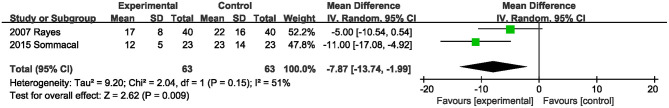
Effect of probiotics or synbiotics supplementation on the length of hospital stay.

Data on the hospital stay were described in two studies (20, 22). Probiotics or synbiotics significantly reduced the hospital stay length compared with the control group (MD, −7.87; 95% CI, −13.74, −1.99; *P* = 0.009; *I*^2^ = 51) (Figure 6).

#### Days of Antibiotic Usage

**Figure 7:**
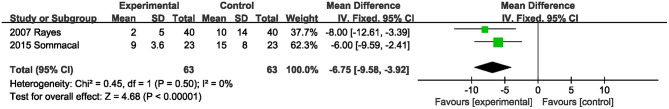
Effect of probiotics or synbiotics supplementation on the antibiotic duration.

Two studies (20, 22) evaluated the effect of probiotics or synbiotics supplementation on the antibiotic duration. Probiotics or synbiotics significantly shortened the duration of antibiotic use (MD, −6.75; 95% CI, −9.58, −3.92; *P* < 0.00001; *I*^2^ = 0) (Figure 7).

### Sensitivity Analysis

The results showed that excluding any one study did not affect the overall effect size of the postoperative infection incidence. The total effect size for perioperative mortality changed when the study by Rayes et al. (20) or the study by Yokoyama et al. (23) was excluded. Sensitivity analysis indicated that the study by Sommacal et al. (22) prominently affected the total effect size of delayed gastric emptying.

### Publication Bias

Egger's test results did not indicate potential publication bias of postoperative infection (*P* = 0.902) and perioperative mortality (*P* = 0.519).

## Discussion

To our knowledge, this is the first meta-analysis exploring the effects of probiotics or synbiotics on post-pancreaticoduodenectomy complications. The RCT evidence indicates that while probiotic or synbiotic supplementation does not reduce the mortality rate after pancreaticoduodenal surgery, it does reduce the incidence of postoperative infections and delayed gastric emptying and also shortens the durations of hospital stay and antibiotic usage. These results are clinically important, as shorter hospital stays and antibiotic administration periods reduce the incidences of antibiotic resistance and iatrogenic infection.

Studies in recent years have shown that probiotics and synbiotics have potential benefits for reducing surgery-related complications (27). Rayes et al. (28) found that synbiotic supplementation reduced the risk of infectious complications after liver transplantation. Probiotic supplementation also improved clinical outcomes in patients undergoing colorectal surgery by lowering inflammatory cytokine levels, reducing the incidence of postoperative infections, changing the tumor microenvironment, and shortening the antibiotic administration duration (29). Further, a meta-analysis by Chowdhury et al. (14) showed that probiotics and synbiotics reduced elective abdominal surgery-associated complications. However, significant differences were found in the postoperative morbidity and mortality among the surgery types. Therefore, it is necessary to explore the preventative effects of probiotics and synbiotics against various surgical complications, especially those with high morbidity and mortality. Few studies have examined the effects of probiotics and synbiotics on the short-term surgical outcomes of pancreaticoduodenectomy; these studies, such as the ones by Rayes et al. (20) and Diepenhorst et al. (21), have reported conflicting results. Thus, a review of the current evidence regarding prebiotic and synbiotic supplementation during pancreaticoduodenectomy is essential.

Pancreaticoduodenectomy is a highly invasive surgical procedure, and several studies investigating the use of probiotics or synbiotics for the prevention of postoperative complications following similar highly invasive procedures have confirmed our results. Rammohan et al. (30) found that synbiotics reduced the antibiotic therapy duration and risk of sepsis and shortened the hospital stay in patients with pancreatitis undergoing pancreatectomy. The results of Sugawara et al. (31) suggest that synbiotic supplementation enhances the immune response, thereby reducing inflammation and the risk of postoperative infection in patients undergoing surgery for biliary carcinoma. Additionally, synbiotic supplementation was noted to reduce the risk of infection after hepatopancreatobiliary surgery by 73% (32). Interestingly, a systematic review noted that synbiotics may not reduce infectious complications after pancreaticoduodenectomy (33); however, this review included only two studies (20, 23). The differences between the results of this review and that of the present meta-analysis may result from the inclusion of four more recent studies in the latter (5, 21, 22, 26).

Subgroup analysis showed that either probiotic or synbiotic supplementation reduced the incidence of infection after pancreaticoduodenectomy. This result is similar to that of Chowdhury et al. who conducted a meta-analysis and found that probiotics and synbiotics were effective strategies for preventing infections after elective abdominal surgery and that bibiotics were more effective than probiotics (14). Our study did not find that synbiotics were superior to probiotics. Therefore, future studies should focus on the best alternative treatment (i.e., probiotics or synbiotics) to reduce the incidence of infections after pancreaticoduodenectomy.

Postoperative infections and delayed gastric emptying generally prolong hospital stay and antibiotic usage (1). Therefore, the shortened hospital stay and antibiotic therapy period may be related to the reduced incidence of postoperative infections and delayed gastric emptying due to probiotic and symbiotic supplementation. Additionally, probiotics can promote the recovery of gastrointestinal function (34, 35); a similar observation was made in our study wherein probiotics and synbiotics reduced the occurrence of delayed gastric emptying.

The mechanism underlying the beneficial effects of probiotics and synbiotics on pancreaticoduodenectomy is unclear, but may be related to several factors. First, studies have shown that intestinal flora dysregulation caused by surgical stress increases the risk of postoperative complications. However, probiotics and synbiotics regulate intestinal flora and help restore the normal intestinal microorganism balance, thereby reducing the risk of postoperative complications (36). Second, probiotics can reduce intestinal flora translocation, subsequently reducing the incidence of infection (37). Further, probiotics and synbiotics protect the intestinal mucosal barrier, maintain normal intestinal mucosal permeability, and reduce toxin absorption (38–40). Finally, probiotics regulate innate and adaptive immune responses and enhance local immune function (27).

Probiotics and synbiotics have been used for decades, and numerous studies have demonstrated that they are safe (41). A meta-analysis (42) suggested that probiotics do not increase mortality in critically ill patients and that probiotics and synbiotics are well-tolerated in patients with significant immunosuppression, such as those undergoing a major gastrointestinal reconstructive surgery or liver transplantation (14). However, in the present meta-analysis, none of the included studies described probiotic or synbiotic safety. Considering that pancreaticoduodenectomy is a highly invasive procedure, the assessment of adverse effects should be explored in future studies.

Our study has three main advantages. First, a comprehensive literature search was conducted with no filters, thus reducing potential bias. Second, we set strict inclusion criteria and only analyzed the RCTs that met these criteria to ensure the reliability of our results. Finally, advanced statistical methods demonstrated the robustness of our conclusion.

Conversely, this meta-analysis has four limitations. First, only six RCTs were included, and all comprised a small number of subjects. Second, different probiotics and synbiotics were used among the studies, and it was not possible to determine which probiotics and synbiotics were the most effective. We found that most of the studies used *Lactobacilli spp*. Therefore, we speculate that *Lactobacilli* as probiotics have the most benefits, and recommend the same to be the focus of future studies. Furthermore, the effect of probiotics on the hospital stay and antibiotic usage duration was based on a pooled analysis of results from a small number of studies. Finally, only two studies used a double-blinded design, which could lead to potential bias.

In conclusion, this study demonstrated the value of probiotic or synbiotic supplementation in patients undergoing pancreaticoduodenectomy, as evidenced by the reduced incidence of infectious complications and delayed gastric emptying. Further, the hospital stays and antibiotic administration periods were shorter. Our results highlight the importance of probiotics or synbiotics for healthcare systems, and offer a potential strategy for preventing complications and promoting recovery after pancreaticoduodenectomy, thereby saving medical resources and reducing the burden on healthcare. However, because limited studies have been performed to date, these results remain questionable and should be interpreted with caution considering the study limitations. Multicenter, large-sample RCTs are necessary to validate the effect of probiotics or synbiotics on the clinical outcomes of pancreaticoduodenectomy.

## Data Availability Statement

The original contributions presented in the study are included in the article/supplementary material, further inquiries can be directed to the corresponding authors.

## Author Contributions

ZW, LZ, GT, and JT conceptualization and writing—review and editing. LZ, GT, and JT data collection and analyses. GT and LZ writing—original draft preparation. ZW, LZ, GT, and JT had primary responsibility for final content. All authors contributed to the article and approved the submitted version.

## Conflict of Interest

The authors declare that the research was conducted in the absence of any commercial or financial relationships that could be construed as a potential conflict of interest.

## Publisher's Note

All claims expressed in this article are solely those of the authors and do not necessarily represent those of their affiliated organizations, or those of the publisher, the editors and the reviewers. Any product that may be evaluated in this article, or claim that may be made by its manufacturer, is not guaranteed or endorsed by the publisher.
